# Supplementary material for: The Assessment of Skin Homeostasis Changes after Using Different Types of Excipients in Healthy Individuals
Source: Int J Environ Res Public Health. 2022 Dec 12;19(24):16678. doi: 10.3390/ijerph192416678 (PMC9778961; doi:10.3390/ijerph192416678)
Supplement: Supplementary file 1 [file ijerph-19-16678-s001.zip › ijerph-2047194-supplementary.pdf]

Table S1. CONSORT 2010 checklist of information to include when reporting a within-person randomised trial. For within-person trials, a group is the set of participants' body sites that was allocated a particular intervention.

Section/Topic	Item no.	Standard CONSORT Checklist item	Extension for within-person trials	Page no.
**Title and abstract**				
	1a	Identification as a randomised trial in the title	Identification as a within-person randomised trial in the title	1
	1b	Structured summary of trial design, methods, results, and conclusions (for specific guidance see CONSORT for abstracts [3])	Specify a within-person design and report all information outlined in table 2	1
**Introduction**				
Background and objectives	2a	Scientific background and explanation of rationale		**2**
	2b	Specific objectives or hypotheses		3
**Methods**				
Trial design	3a	Description of trial design (such as parallel, factorial) including allocation ratio	Rationale for using a within-person design and identification of body sites	3
	3b	Important changes to methods after trial commencement (such as eligibility criteria), with reasons		3
Participants	4a	Eligibility criteria for participants	Eligibility criteria for body sites	3
	4b	Settings and locations where the data were collected		4
Interventions	5	The interventions for each group with sufficient details to allow replication, including how and when they were actually administered	Whether interventions were given sequentially or concurrently	4
Outcomes	6a	Completely defined pre-specified primary and secondary outcome measures, including how and when they were assessed	Outcomes should be clearly defined as per-site or per-person	4

Section/Topic	Item no.	Standard CONSORT Checklist item	Extension for within-person trials	Page no.
	6b	Any changes to trial outcomes after the trial commenced, with reasons		4
Sample size	7a	How sample size was determined	Report the correlation between body sites	4
	7b	When applicable, explanation of any interim analyses and stopping guidelines		4
Randomisation:				
Sequence generation	8a	Method used to generate the random allocation sequence		4
	8b	Type of randomisation; details of any restriction (such as blocking and block size)	Methods used to determine the allocation sequence of body sites and treatments within an individual (e.g. how first site to be treated was decided)	4
Allocation concealment mechanism	9	Mechanism used to implement the random allocation sequence (such as sequentially numbered containers), describing any steps taken to conceal the sequence until interventions were assigned		4
Implement-ation	10	Who generated the random allocation sequence, who enrolled participants, and who assigned participants to interventions	Replaced by 10a	4
	10a		Who generated the random allocation sequence, who enrolled participants, and who assigned body sites to interventions	4
Blinding (masking)	11a	If done, who was blinded after assignment to interventions (for example, participants, care providers, those assessing outcomes) and how		4
	11b	If relevant, description of the similarity of interventions		4

Section/Topic	Item no.	Standard CONSORT Checklist item	Extension for within-person trials	Page no.
Statistical methods	12a	Statistical methods used to compare groups for primary and secondary outcomes	Statistical methods appropriate for within-person design	5
	12b	Methods for additional analyses, such as subgroup analyses and adjusted analyses		5
**Results**				
Participant flow (a diagram is strongly recommended)	13a	For each group, the numbers of participants who were randomly assigned, received intended treatment, and were analysed for the primary outcome	Number of participants and number of body sites at each stage	5
	13b	For each group, losses and exclusions after randomisation, together with reasons	Number of participants and number of body sites lost or excluded at each stage, with reasons	5
Recruitment	14a	Dates defining the periods of recruitment and follow-up		5
	14b	Why the trial ended or was stopped		5
Baseline data	15	A table showing baseline demographic and clinical characteristics for each group	Baseline characteristics for site and individual participants as applicable	5
Numbers analysed	16	For each group, number of participants (denominator) included in each analysis and whether the analysis was by original assigned groups	Number of randomised body sites in each group included in each analysis	5
Outcomes and estimation	17a	For each primary and secondary outcome, results for each group, and the estimated effect size and its precision (such as 95% confidence interval)	Observed correlation between body sites for continuous outcomes and tabulation of paired results for binary outcomes	6
	17b	For binary outcomes, presentation of both absolute and relative effect sizes is recommended		6

Section/Topic	Item no.	Standard CONSORT Checklist item	Extension for within-person trials	Page no.
Ancillary analyses	18	Results of any other analyses performed, including subgroup analyses and adjusted analyses, distinguishing pre-specified from exploratory		6
Harms	19	All important harms or unintended effects in each group (for specific guidance see CONSORT for harms)	Harms or unintended effects reported by participant and by body site	6–7
**Discussion**				
Limitations	20	Trial limitations, addressing sources of potential bias, imprecision, and, if relevant, multiplicity of analyses		9
Generalisability	21	Generalisability (external validity, applicability) of the trial findings		8
Interpretation	22	Interpretation consistent with results, balancing benefits and harms, and considering other relevant evidence		8
**Other information**				
Registration	23	Registration number and name of trial registry		-
Protocol	24	Where the full trial protocol can be accessed, if available		-
Funding	25	Sources of funding and other support (such as supply of drugs), role of funders		9

Table S2. Measure values for excipients and parameters: TEWL, transepidermal water loss; SCH, stratum corneum hydration; AU; arbitrary units; W/O, water-in-oil; O/W, oil-in-water. p-value after using Student T-tests or Mann-Whitney tests between control and other excipient. To obtain P-value inc/drec we used Friedman test a ANOVA followed by an α-adjusted post hoc Bonferroni test.

	**Basal**	**Control 10 min**		
		**Control 10 min**	**C-C10**	***p*-value**
**Temperature**	29.84 °C (± 3.9)	36.46 (± 1.55)	−6.63	0.193
**pH**	5.96 (± 1.01)	6.24 (± 0.55)	−0.28	0.002
**Melanin**	103.45 AU (± 93.38)	96.89(± 80.26)	6.56	0.249
**Erythema**	223.63 AU (± 65.2)	219.99(± 68.34)	3.64	0.531
**TEWL**	11.28 g·m^{-2}·h^{-1} (± 8.12)	9.94 (± 5.93)	1.33	0.281
**SCH**	45.4 AU (± 12.89)	46.05(± 11.78)	−0.64	0.5
**R0**	0.06 mm (± 0.03)	0.06(± 0.03)	0	0.881
**R2**	65.94% (± 10)	68.84% (± 12)	−2.9	0.041
**R5**	54.64% (± 18)	56.69% (± 20)	−2.1	0.241
**R7**	44.86% (± 13)	47.15% (± 15)	−2.6	0.056

		**W/O**	
	**W/O**	**C-W/O**	***p-value***
**Temperature**	30.46 °C (± 1.28)	−0.62	0.221
**pH**	6.08 (± 0.85)	−0.12	0.068
**Melanin**	112.07 AU (± 88.32)	−8.62	0.232
**Erythema**	198.62 AU (± 64.71)	25.01	<0.001
**TEWL**	9.69 g·m^{-2}·h^{-1} (± 4.15)	1.58	0.18
**SCH**	43.3 AU (± 9.96)	2.1	0.303
**R0**	0.06 mm (± 0.02)	0	0.899
**R2**	69.82% (± 10)	−3.00	0.023
**R5**	60.95% (± 18)	−6.30	<0.001
**R7**	50.88% (± 14)	−6.00	<0.001

		**O/W**	
	**O/W**	**C-O/W**	**p-value**
**Temperature**	30.79 °C (± 1.08)	−0.95	0.068
**pH**	5.86 (± 0.74)	0.11	0.123
**Melanin**	119.19 AU (± 83.05)	−15.73	0.036
**Erythema**	184.27 AU (± 66.72)	39.36	<0.001
**TEWL**	8.40 g·m−2·h−1 (± 2.8)	2.87	0.009
**SCH**	52.25 AU (± 8.4)	−6.85	< 0.001

**R0**	0.06 mm (± 0.02)	0	0.846
**R2**	67.02% (± 11)	−1.10	0.428
**R5**	61.15% (± 19)	−6.50	<0.001
**R7**	49.66% (± 13)	−4.80	<0.001
		**Beeler**	
	**Beeler**	**C-Beeler**	**p-value**
**Temperature**	30.91 °C (± 1.15)	−1.07	0.038
**pH**	5.97 (± 0.61)	−0.01	0.957
**Melanin**	126.38 AU (± 86.52)	−22.93	0.005
**Erythema**	182.4 AU (± 67.34)	41.23	<0.001
**TEWL**	8.75 (± 2.62)	2.53	0.021
**SCH**	61.32 AU (± 12.31)	−15.92	<0.001
**R0**	0.05 mm (± 0.02)	0.01	0.012
**R2**	69.03% (± 1)	−3.10	0.037
**R5**	65.82% (± 25)	−11.20	0.001
**R7**	52.04% (± 12)	−7.20	<0.001
		**Foam**	
	**Foam**	**C-Foam**	***p-value***
**Temperature**	31.24 °C (± 1.17)	−1.41	0.007
**pH**	6 (± 0.46)	−0.04	0.661
**Melanin**	126.43 AU (± 85.34)	−22.98	0.002
**Erythema**	201.63 AU (± 66.06)	22	0.001
**TEWL**	7.41 $g \cdot m^{-2} \cdot h^{-1}$ (± 1.83)	3.86	0.001
**SCH**	40.73 AU (± 11.24)	4.68	0.004
**R0**	0.05 mm (± 0.02)	0.01	0.026
**R2**	71.33% (± 1)	−5.40	<0.001
**R5**	65.32% (± 15)	−10.70	<0.001
**R7**	55.29% (± 12)	−10.40	<0.001
		**W/O**	

	W/O		C-W/O	*p-value*
**Temperature**	30.46 °C (± 1.28)		−0.62	0.221
**pH**	6.08 (± 0.85)		−0.12	0.068
**Melanin**	112.07 AU (± 88.32)		−8.62	0.232
**Erythema**	198.62 AU (± 64.71)		25.01	<0.001
**TEWL**	9.69 $g \cdot m^{-2} \cdot h^{-1}$ (± 4.15)		1.58	0.18
**SCH**	43.3 AU (± 9.96)		2.1	0.303
**R0**	0.06 mm (± 0.02)		0	0.899
**R2**	69.82% (± 10)		−3.00	0.023
**R5**	60.95% (± 18)		−6.30	<0.001
**R7**	50.88% (± 14)		−6.00	<0.001
		**Vaseline**		*p-value inc/dec*
	**Vaseline**	**C-Vaseline**	*p-value*	
**Temperature**	31.13 °C (± 1.74)	−1.29	0.017	< 0.001
**pH**	6.4 (± 0.43)	−0.44	<0.001	0.091
**Melanin**	129.9 AU (± 84.16)	−26.45	<0.001	0.294
**Erythema**	182.63 AU (± 68.26)	41	<0.001	<0.001
**TEWL**	7.38 $g \cdot m^{-2} \cdot h^{-1}$ (± 2.33)	3.9	<0.001	0.011
**SCH**	17.81 AU (± 13.44)	27.59	<0.001	0.155
**R0**	0.06 mm (± 0.02)	0	0.287	0.57
**R2**	75.65% (± 11)	−9.70	<0.001	<0.001
**R5**	66% (± 16)	−11.40	<0.001	<0.001
**R7**	55.71% (± 0.13)	−10.80	<0.001	<0.001
